# Dairy Production under Climatic Risks: Perception, Perceived Impacts and Adaptations in Punjab, Pakistan

**DOI:** 10.3390/ijerph16204036

**Published:** 2019-10-21

**Authors:** Qasir Abbas, Jiqin Han, Adnan Adeel, Raza Ullah

**Affiliations:** 1 College of Economics and Management, Nanjing Agricultural University, Nanjing 210095, China; qaisarabbas@uaf.edu.pk (Q.A.); a_adni@uaf.edu.pk (A.A.); 2 Institute of Agricultural & Resource Economics, University of Agriculture, Faisalabad 38000, Pakistan; raza_khalil@yahoo.com; 3 Institute of Business Management Sciences, University of Agriculture, Faisalabad 38000, Pakistan

**Keywords:** climate change, farmers’ risk perceptions, dairy production, perceived impacts, adaptation, Pakistan

## Abstract

The changing climatic conditions coupled with fodder availability have posed severe challenges and threats for the dairy sector in Pakistan. The current paper determines the influence of climate change on the dairy sector in Pakistan. Comprehensive data set was collected from 450 farmers. The majority of farmers experienced the climate change and its variability and explained that severity and frequency of climatic extreme events such as droughts, heat waves, floods, pests and diseases and humidity is increasing. The study found that farmers considered drought as one of the major climatic risks which severely affects all aspects of dairy production. Specifically, to estimate the perceived impacts of climatic extreme event on milk production, an ordered probit model was applied and identified that climate change had high adverse impact on milk quantity in the study area. Different adaptation practices, such as changing cropping pattern for fodder production, off-farm income activities, diversifying the farm and regular vaccination are mostly used by dairy farmers. The study recommends policy initiatives to be taken by government for long term developments in the dairy farming.

## 1. Introduction 

Climate change refers to the long-term changes in temperature, precipitation, wind and other climatic features [1]. The Intergovernmental Panel on Climate Change (IPCC) in its 5th assessment report specifies that by the end of this century, average temperature on the surface of the planet may rise between 0.3 and 4.8 °C [2]. The increasing influence of climate change on crops and livestock rearing systems is more negative in nature [3]. Likewise crops, livestock are also almost sensitive that climate extreme events have extremely affected them in the past [4]. It is obvious that climate change and its severe impacts on productivity of livestock impose additional devastated risks to the worldwide food security. Latest annual report of IPCC (AR5) illustrates that the most tropical regions of world are not adapting the mitigated measures for climate change that exaggerate more negative effects on food production systems [3]. 

Climate change has imposed negative impacts on livestock productivity in addition to adulterated water resources, poor quality of feed and prone diseases of livestock [5,6,7,8,9]. Heat stress in livestock due to rising temperatures leads to an adverse impact on milk production [10,11], reproduction [12], health [13] and mortality rate of animals [14]. According to Houghton (2001) and Herbut (2018) [15,16] and Rust (2013) [17] air temperature, humidity and wind speed have significant direct effect on milk production and reproduction rate. Due to the persistent drought conditions, the lactation period of dairy cattle’s always shrinks. Likewise, milk production quantity and quality declines [18]. In Pakistan, livestock production is likely to reduce by 20 to 30% in the future because of increasing temperature spells. Ultimately, a crisis in dairy and meat industry will increase the prices of products that will not be in the reach of middle class consumers [19].

Pakistan is the state that have also been adversely influenced because of climate change, its vulnerability, low adaptive capacity of mitigating strategies and poor infrastructure at farm level [2]. There is future prediction that by the end of 2050, the temperature in Pakistan will increase by 2–3 °C, and the precipitation rate will also be significantly varied [20]. The Global Climate Risk Index (GCRI) ranks Pakistan at 8th position among the countries the most affected by climate change and extreme weather events from 1995 to 2014 [21].

As the livestock sector is the central pillar of Pakistan’s rural economy, it contributes around 60.5% in value addition of agriculture as well as 11.4% in overall Gross Domestic Product (GDP). The most marginal rural people consider livestock sector as the best source of income. More than eight million rural families are directly involved in raising livestock and derive up to 35% of their livelihoods from livestock [22]. The livestock sector is playing a vital role in the mitigation of poverty and in the form of gross value addition, enhancing 1333 billion rupees (8.495 billion USD) as foreign exchange reserves. Climate change and poor endowments of natural resources are the most significant development challenges confronting the dairy farming in Pakistan. Small and marginal farmers’ share in milk production is more than 70% and they are highly susceptible to unpredictable events of extreme weather [23].

Climate change is the main cause of weather-related hazards that adversely affect livestock production system especially in developing countries. Policy makers and researchers agree at this point that climate change significantly impact the livestock sector [5,24]. Unfortunately, in South Asia, limited research on potential impacts of climate related risks on livestock sector is available compared to the cropping sector [25,26]. 

Farmers’ activities and plans to take adaptation measures are more dependent on their perceptions about climate variability and risks as compared to actual climate scenarios [27,28,29]. Perception insights could be economical and technically feasible to design the comprehensive policies related to climate change and environmental protection [30,31,32]. Moreover, climate-related risk perceptions enable farmers to decide for effective adaptation of risk coping strategies [33,34,35,36].

**Figure 1:**
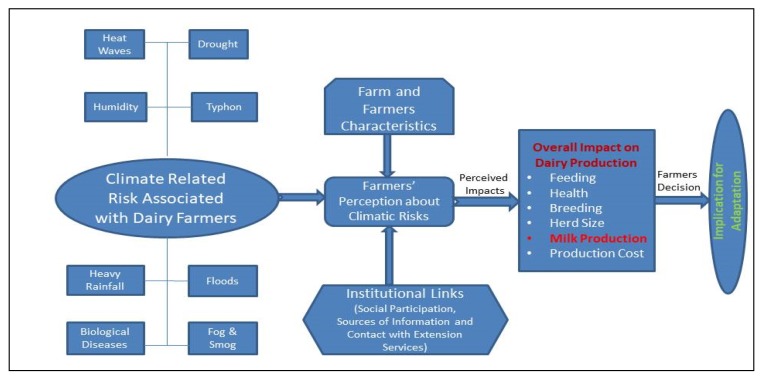
Dairy farming context under climatic risks.

Figure 1 illustrates the conceptual framework. The basic theme behind this conceptual framework is that climate change impact can be quantified by farmers’ perceptions and their capability to adopt the most effective risk coping strategies [37,38].

Considering the importance of livestock for small and marginal farmers, determination of perceived impacts of climate change on different aspects in dairy farming will be helpful to formulate adaptation policies for forthcoming challenges. Research studies in Pakistan on this topic are limited, so there is dire need to identify the perceptions of farmers about climatic risks and its influence on dairy production generally and on milk production specifically. Mostly, dairy farmers depend upon the pasture and crop residues for animal feeding in the South Asian region. Therefore, observational insights are deemed essential to identify the adaptation or coping strategies practiced by farmers, to reduce the climate related risks for their dairy farming system.

The main aim of the current study is to investigate the implications of the climate change on livestock productivity in Punjab, Pakistan including key sub-objectives as (1) to assess dairy farmers’ perceptions about climate related risks and its variability, and (2) to measure the perceived impact of climate related risks on dairy production systems, especially milk production. 

This research article is organized in the following sections. Research data and methods are presented in Section 2. Furthermore, empirical results of the study are given in Section 3 while Section 4 has a detailed discussion of results. The last section concludes the study with some policy implications.

## 2. Material and Research Methods

### 2.1. Study Area

**Figure 2:**
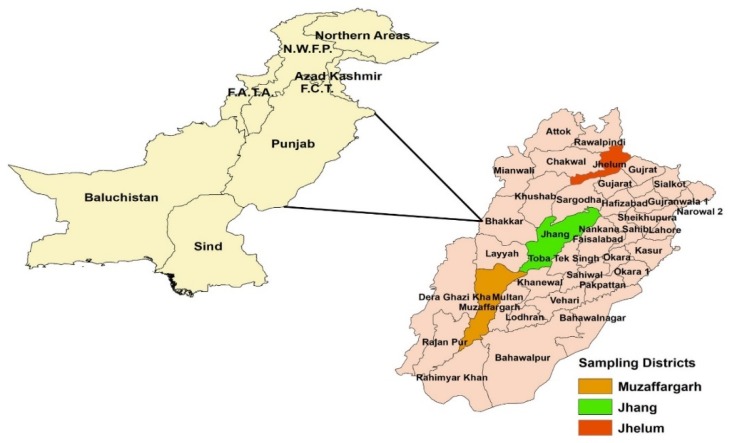
Map of study area.

The research was carried out in the Punjab, which is the second largest and the most populated province in Pakistan and located in the semi-arid low land region [39]. The geographical area of Punjab has 20.63 million hectares, in which 59% area is under cultivation. Reason being selecting this province includes that Punjab contributes its share of 54% and 62% in the national GDP and national agricultural sector [40]. Punjab has the largest buffalo and cow population, at 16.019 million and 13.204 million in total livestock population, respectively. Punjab is also the major milk producing province with annual production of 36.23 million liters, which is about 67% of total milk production in Pakistan [22]. Mixed cropping along with livestock rearing is the common practice of farmers in Punjab [41,42]. In Punjab, the average minimum and maximum temperature per annum has been in range of 16.52 °C to 21.50 °C and 30.09 °C to 32.75 °C between the periods 1980–2018. In Punjab, 50% to 75% rainfall is linked with the monsoon season that lasts from June to August each year. The Pakistan Agriculture Research Council (PARC) map bifurcates Punjab into different agro-ecological zones, as depicted in Figure 2, and each zone has different rainfall pattern. Rain-fed and irrigated zones of Punjab receives the highest and lowest rainfall respectively [43]. Keeping this in mind, three districts from three different agro-ecological zones were selected for purposes of this study.

Most of the farmers in selected districts grow crops as well as rear livestock as the second best source of income. The rain-fed area of Punjab has the lowest number of dairy animals as we can see from Table 1. 

### 2.2. Sampling Procedure and Data Collection Methods

**Figure 3:**
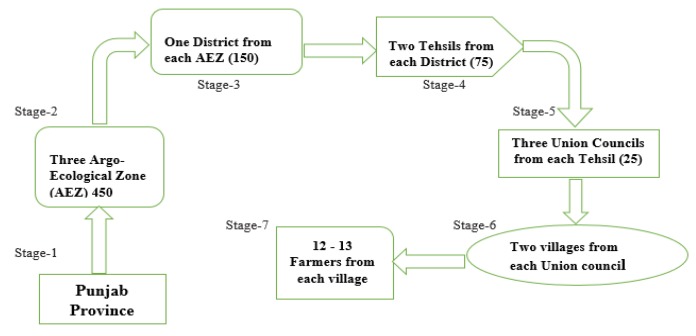
Sampling framework.

A field survey was carried out to collect cross sectional data from September to December 2018. A well designed and structured topic guide/questionnaire was used to draw the valuable data by interviewing dairy farmers/respondents. A multi-stage stratified random sampling procedure was applied as shown in Figure 3. In the first stage, study region was selected and for this purpose, map of PARC indicating agro-ecological zones of Punjab province was used. The Punjab province is divided into four agro-ecological zones which are irrigated plains (low intensity rain-fed area), mixed cropping zone (moderate rain-fed area), Barani (high intensity rain-fed) region and Thal region (desert area). Researchers selected the first three zones. In the second stage, total three districts consisting of one district from each zone by considering the homogeneity and heterogeneity of climatic variability, cropping pattern and irrigation systems and cattle breed were selected. From each district, two random cities (tehsils/sub-district) were selected at third stage and in next stage, from each city; three union councils (UCs) were chosen for study purpose by considering distance between UCs and distance from the UC to the main city. According to the Government of Pakistan administrative system, each UC has some villages and in the last fifth stage, from each UC, two villages were chosen. Twelve to thirteen dairy farmers were approached for interview without considering the size of their land holdings and farm location. On the whole, 450 farmers and out of which 150 were interviewed from each selected district. 

### 2.3. Data Analysis

#### 2.3.1. Climatic Risk Perception

During the interview, respondents were probed about the awareness of climate change and the severity and intensity of different climate related risks. Questions regarding climate related extreme events and fluctuations in climate patterns were asked by answering categorically in the form of increasing, no change and decreasing during the last decade (2008–2018). The following relevant categories: (1) Animal health (2) Feeds and feeding (3) quality and quantity of milk product (4) Animal breeding (5) Herd Size and (6) Production cost were developed to determine the farmers’ perceived impact of climate change on cattle production. Likewise, a Likert scale from 1 (no impact at all) to 5 (very high impact) was used to determine the total influence on milk production. Descriptive statistics were applied to assess their perceptions about impacts of climate change. The graphs were developed by summarizing the frequencies of responses in%ages.

#### 2.3.2. Empirical Model 

On the basis of utility maximization theory, Random Utility Model (RUM) states that a choice decision may be made in which an individual *i* can choose among a set of alternative j [45]. In this regard, the j ordered categories were developed by five point Likert scale in the form of 1 with no impact on milk production, 2 with low, 3 with medium, 4 with high and 5 with very high impact. An individual farmer chooses the best alternative from a set of choices that maximizes utility. Moreover, the level of farmer’s utility is a function of socio-economic characteristics. An ordered probit model (discrete choice model) was applied to evaluate the factors influencing farmers’ perceptions of climate related risks on milk production in comparison with linear model. Because the values of dependent variable are not continuous in nature but discrete and ordered [46] and it also avoided to detect a non-existing effect as well as loss of power problem like fail to detect an effect [47]. The specified model is given in Equation (1).

(1) $$M\overset{\times }{P}i=\overset{14}{\underset{i=1}{\sum }}X_{i}\beta \;+\;\epsilon_{i}\;\;\;\;\;\;\;\;\;i=1,\;2,\ldots \ldots \ldots ..,\;n$$

MP_i_^*^ is the latent unobserved variable that corresponds perceived milk production affected by climate related risks, *X_i_* is the vector of observed explanatory variables (climatic parameters, farmers’ and farm characteristics and institutional links) of the *i*th farmer, the unknown parameter is estimated as β while the random term of the latent utility function is denoted as $\epsilon_{i}$. The details of the model are given in the Appendix A.

## 3. Estimated Results 

### 3.1. Dairy Farmer’s Socio-Economic and Farm Characteristics

Table 2 presents the socio-economic and farm characteristics of the sampled farmers. The farmer’s average age was around 45 years, while the mean dairy farming experience was of 21 years. It is clear that ranchers were elderly with extensive dairy experience. The growing age of Pakistani farmers is becoming a concern for the agricultural sector [48]. Some researchers use age as proxy variable of agricultural experience [49] and some believe that farmers observe climate changes definitely as their farming knowledge increases [50].

The family size of the respondents was large as main rural families of Pakistan lived in joint family structures [9]. The average size of family members was 8 with 3.6 family members’ variation. Dairy farmers earn about 42% of their income via dairy farming which is major one and the daily-based source of income, followed by crops, labor and public and private sector employment. These figures, confirmed by the Government of Pakistan in 2018, estimates that farmers derive 35% of their annual income from the livestock sector. Around 69% of the respondents are literate at school level, while only 11% attended the college or university and rest (20%) has never been in school. 

The average farm size was 2.9 hectare and holds 4 to 5 milking animals with 14 L milk production per day which varied about 6.5 L due to different reasons. About 52% and 47% of the farms were located in irrigated region and rain-fed area of Punjab province respectively. Most of the dairy farmers (51%) have mixed breed of animals and 38% of farmers are still relying on indigenous/dessi breed, while only 11% are rearing crossbreed animals. Farmers believe that indigenous breeds are more resilient to climate related risks [51].

### 3.2. Dairy Farmers’ Perceptions about Climatic Risks and Variability

**Figure 4:**
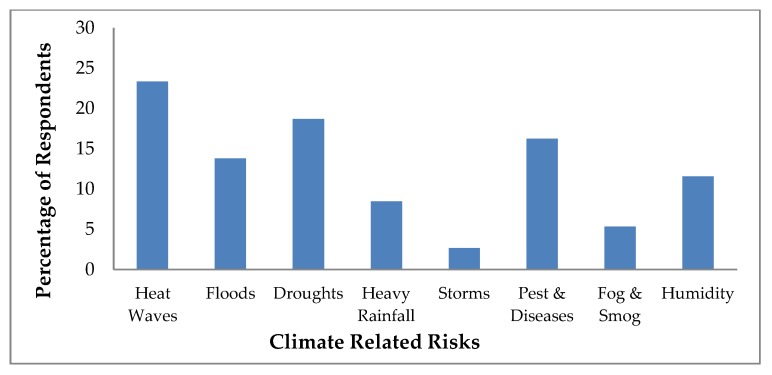
Climate-Related risks perceived by the dairy farmers.

During the interview, dairy farmers were also queried about their perceptions that how they associate different sources of climate related risks with their regular farming practices as presented in Figure 4. Dairy farmers perceived that heat waves were the major threat for their dairy animals followed by droughts, pest and diseases, flood and humidity. About 105 (23%) farmers consider heat waves as one of the major climate related risk sources. Because temperature range (maximum and minimum) in Pakistan is increasing day by day, that severely affects the farming system. 

The second major risk was drought, which also adversely impacts the livestock and fodder production system. Around 73 (16%) respondents reported that animals’ diseases’ severity and sensitivity has increased over the time period and animal health and their productivity and reproduction cycle has altered due to number of biological diseases emerged by fluctuating climatic conditions. Fourteen% of dairy farmers consider flood a significant risk due to a series of floods from 2010 to 2014. Nearly 12% and 8% of farmers identified humidity and heavy rainfall as major climate related risks that affect the dairy production system in negative manner.

**Figure 5:**
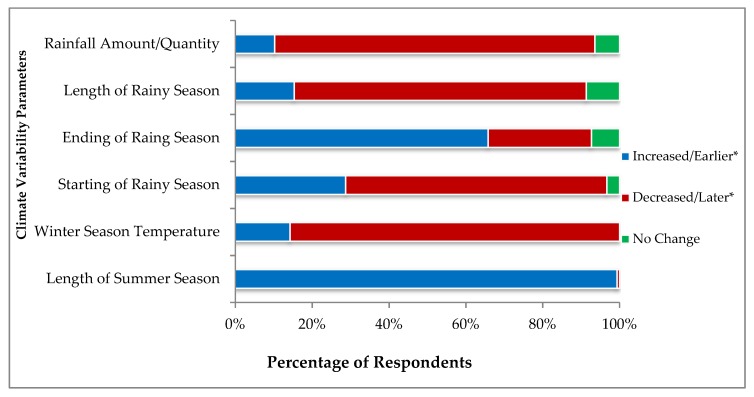
Climatic variability perceived by the dairy farmers.

The farmer’s perception about climate variability is observed as overall understanding of the following climate parameters. During the survey, the question was raised to the dairy farmers as to whether they have perceived some fluctuations in precipitation and temperature during last decade and either the length of summer season has increased, decreased or there has been no change during the last decade. Simultaneously, the same approach was used for getting perceptions about the temperature in the winter season, while for the rainy season, we inquired that either monsoon has been started earlier, later or continuing on the same pattern during the said time period. All the responses reported by the surveyed farmers are summarized in Figure 5.

It was a common perception of almost all farmers that the time interval in summer season had been extended. More than 85% farmers reached on the consensus that winter season temperature is increasing that means the frequency and intensity of winter has been decreased in the Punjab province. Around 25% of farmers observed that raining season has been starting earlier every year while 70% argued that it starts later and only 5% observes that there is no change in precipitation pattern.

On the other hand, 66% of farmers observed that monsoons period terminate earlier but 27% had conflicts in their views about ending of rainy season. Less than 20% of the dairy farmers were of the view that length of rainy season has increased and almost 75% considered that span of rainy season has decreased and 8% respondents observe no change in length of season. Most of the farmers (85%) observed that the amount/quantity of rainfall has been decreased over the period of the last 10 years.

**Figure 6:**
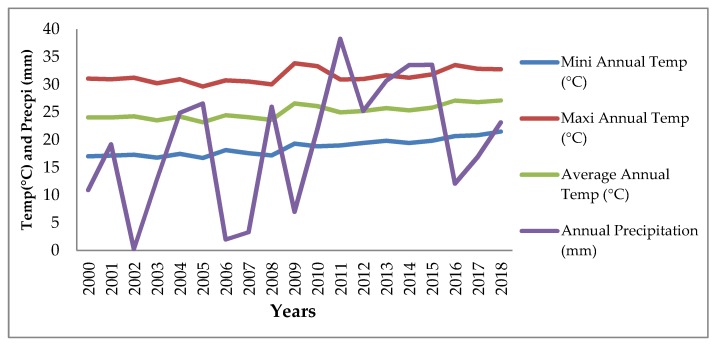
Historical trend of climate change in Punjab, Pakistan Source: Pakistan Meteorological Department (PMD) Dataset.

Figure 6 verifies the perception of the farmers, as we can see that since 2000 onward temperature has an increasing trend with minor variation while the precipitation has too much variation which very disturbing for the dairy farmers as heavy rainfall cause of floods and very low precipitation increased the drought time span. On the whole, we can conclude that farmers correctly perceived the changes in climate. 

### 3.3. Perceived Impacts of Climatic Risks on Dairy Production System

**Figure 7:**
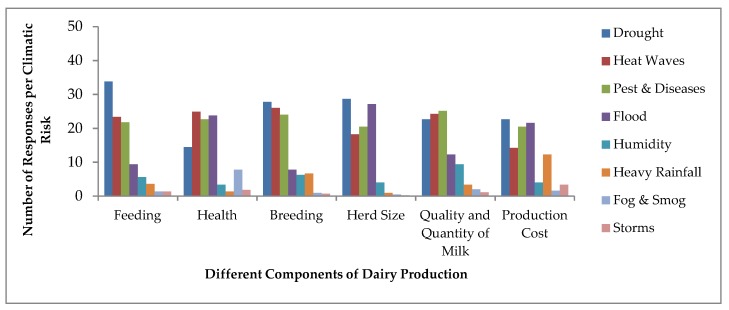
Perceived impacts of climatic risks on dairy farming system.

We measured the perceived impact of pre-defined climate related risks on some aspects (feeding of animals, health of animals, breeding of animals, herd size, milk quantity and quality and production cost) of dairy production system and results are presented in Figure 7 Almost all respondents (98%) reported that climate change has affected their overall dairy production system during last decade. One of the main climate related risks is drought, which affected every aspect of the dairy production significantly. Dairy farmers perceive that heat waves and pests and diseases also posed severe adverse impact on all components of dairy farming. More than 20% of farmers reported that whenever they experienced with floods, ultimately herd size, health and production cost was hurtled. Dairy farmers revealed that during floods, liver fluke infection is the main disease observed in dairy animals.

Most of the respondents believe that during heat stress, milking animals collapse with mastitis disease. During a humid period, milk production decreased noticeably and breeding of animals is also affected. Many farmers observe that during humidity, conception rate in animals is declined and also have the opinion that during heat stress and dry period female cattle are not ready for mating process, which disturbed pregnancy and reproduction rate. About 7% of farmers also recalled that heavy rainfall in unexpected time disturb the heated animals for mating purpose. Heavy rainfalls increased the cost of production at the farm because they bear some unexpected expenditures during continuous rainfall and not able to perform the routine activities for animals. Fog and smog affect the health of animals significantly that is one of the main aspects of dairy farming system. Farmers were on the view that almost all climatic risks increase their production costs because they invested massively to maintain milk production and buy some extra equipments at farm level.

### 3.4. Estimates of Ordered Probit Model

We used an ordered probit model to evaluate perceived impacts of climate related risks on milk production at the farm level. This robust model was tested with specific tests to check the possibility of multicollinearity, heteroscedasticity and goodness of fit [52,53]. Variance Inflation Factor (VIF) was applied for multicollinearity and showed that multicollinearity did not exist in the model. Value of Chi square was highly significant for log likelihood function and coefficients of all explanatory variables were significantly differed from zero. The link test showed that the model was correctly specified.

Wollni (2010) [54] suggested that ordered probit model coefficients are very complex to explain. Estimated parameters of explanatory variables in model only show the direction (positive or negative) of impact on dependent variable and do not measure the actual change in magnitude or probabilities. Thus, marginal effects of the model are however easy to interpret the results. From the five perception levels, ‘no impact’, ‘low’, ‘medium’, high’ and ‘very high’ impact on milk production; we estimated marginal effects for only first four categories because only five farmers out of 450 respondents chose the last perception ‘high impact’ and estimated results of marginal effect for this category were not significant for all explanatory variables so we did not include it in Table 3. Here, we interpreted the marginal effect estimates as effect of changes in explanatory variables on the expected likelihoods underneath the category (high impact) of dependent variable. 

Overall, 14 predictors were examined in the model and out of which 10 predictors showed significance for perceived impact of climate change. All variables had the positive signs and all climatic risk variables increased the degree of perceived impacts. The probability of perceiving ‘medium’ and ‘high’ impact on milk production increased by 2% and 3% respectively due to drought, while the probability of ‘no impact’ and ‘low’ impact on milk production decreased about 1.4% and 4% respectively as drought risk increased. All the remaining climatic risks indicating the similar probable impact on milk production with minor variation except heavy rainfall where the results are insignificant. 

Education significantly influenced the perception of dairy farmers for climate change effects. Likelihood of perceiving ‘medium’ and ‘high’ impact on milk production increased about 4% and 6% respectively among educated farmers, whereas increase in number of schooling years decreased the probability of ‘no impact’ and ‘low impact’ by 3% and 8%, respectively.

The farmers with good dairy farming experience likely perceive more impact on milk production due to climate change by 1%. While, Addition of 1 year in dairy farming experience reduce farmer’s probability for perceiving ‘low’ or ‘no impact’ on milk production about 0.5% and 0.2% respectively. Although farmer’s age showed negative signal for ‘no impact’ and ‘low impact’, but it was a positive sign for ‘medium’ and ‘high’ impact (indicating that aged farmers’ perceived more about climate change impacts on milk production). However, variable results were statistically insignificant.

The number of milking animals increased the production at farm significantly and enhanced the income of the farmers. If the respondent had more milking animals at farm, probably s/he perceived the more impact (‘medium’ and ‘high’) of climate change on milk production about 5% and 6% respectively. In contrast, the probability of perceiving ‘low’ or ‘no impact’ decreased by 0.7% and 0.3% accordingly. Breed of animals had a significant impact on milk production. As the cross breed of cattle had more productivity of milk as compared to local breed. In selected districts, farmers mostly had indigenous/local breed and they perceived 

The minor influence of climate change on milk production is because the local breed was considered to be resilient against some climatic risks. However, the results of breed of animals’ variable were not statistically significant in the model.

Institutional links had a strong influence on the perceived impact of climatic risks as they think that their role is to disseminate the relevant information to the society. Farmers’ participation in social activities increased the probability of perceiving higher impact on milk production about 1% and its perceiving probability for ‘no’ or ‘low’ impact also decreased with 0.5% and 1.5%, respectively. However, it was a non-significant result. Sources of information had greater role about the farmer’s perception for climate related risks. As the farmers had quick and accurate information, then, they would be able to perceive the severity of impact timely and may try to diminish the negative impact of risks by suitable adaptation practices.

The probability of farmers about climatic risks impact ‘high’ on milk production increased by 2.3%, at the same time the perceiving probability of ‘no impact’ or ‘low impact’ decreased about 1% and 3% respectively. Dairy farmers in regular interaction with agricultural extension officers were well informed in advance about climatic risks and they perceive more accurately as compared to others. Contact/access to veterinary officer or extension officer significantly increased the probability of perceiving ‘higher’ impact on milk production by 5%. On the other hand, they perceived ‘low impact’ about 6.4% if they regularly approaching the extension officers.

### 3.5. Risk Coping Adaptation Strategies 

Perception of climate related risks as a major challenge enable dairy farmers to adapt certain coping strategies [9]. Researchers observe that dairy farmers always try to adapt good measures against the negative impacts of climate change but not in a consistent way [55]. Most of the farmers capitalize their dairy farming as compared to cropping sector heavily affected by climatic extreme events [26,48,56]. Farmers solemnly believe that cropping sector is more sensitive than dairy production. That is why they consider this opportunity as good income source in extreme weather conditions and minimize crop failures. Farmers’ are keen to adapt ex-ante risk coping strategies to reduce the negative impacts of climate related risks because such responses enhance their earnings and stabilize their livelihoods [57]. 

**Figure 8:**
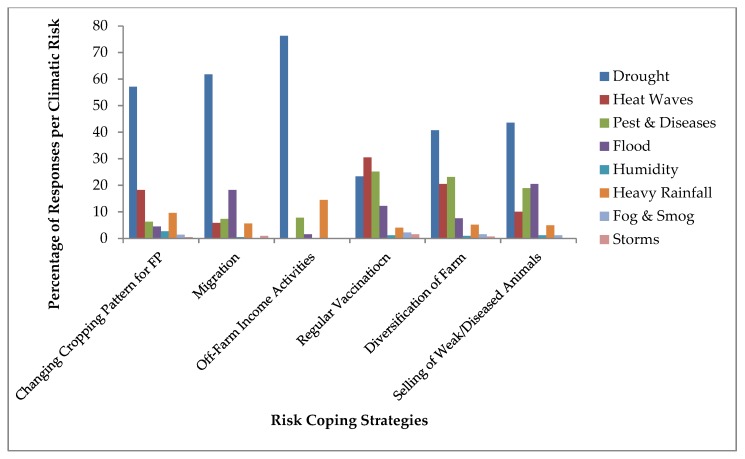
Risk coping adaptation strategies practiced by dairy farmers.

A summary of adaptation strategies of dairy farmers is given in Figure 8. Generally, farmers adopted the following risk coping strategies: changing the cropping pattern for fodder production, migration, off-farm income activities, regular vaccination and diversification of farm and selling of weak/diseased animals. Most of the farmers applied these adaptation practices against drought situation because droughts severely affected their production at farm level.

More than 50% of respondents change the cropping pattern for fodder production during drought period and while more than 15% of farmers also practice this strategy in peak summer and rainy season [39]. About 62% of farmers migrated from one place to other with their herd in the search of fodder and water. During floods some were forced to migrate for their own and animals’ survival [9,58].

Most of the farmers, about 75%, earn an income from selling their labor to non-agricultural activities in cities, in this way they spread the risk and also enables them to invest in their farm in the near future. They diversified their income by working as daily laborer, petty traders, and construction worker or in some industry as wage earner, so income from these activities enables farmers to modernize their production system during unexpected hazards [59,60,61]. Heat waves, pests and diseases, droughts and floods are the major risks which cause the increase of infectious disease and parasite occurrence in cattle. Therefore, farmers use regular vaccination during these climatic risks. Famous livestock breeds and crops cultivars are more resistant against heat waves and droughts. Such strategy of diversification enhances animal productivity during high spells of rainfall and temperature. Furthermore, such diversification in variety of crops and animal species (for meat purpose) is an effective way to combat climate-related diseases and pest outbreaks [7,62,63]. About 45% of farmers sell their animals when they become weak or diseased, particularly during the drought situation when fodder and water shortage increased and they have to buy it from market. Marginal farmers adopt this strategy frequently as they were not able to feed and vaccinate their diseased animals due to lack of financial resources [9].

## 4. Discussion

### 4.1. Perceived Climatic Risks and Variability

In Pakistan, dairy farmers are very much concerned about climatic risks and its variability. Their past experience expose that they are well aware of occurrence and severity of climate. It is difficult to comprehend the nature of climate that poses a major threat for dairy production system as well as livelihoods of farmers [7,64]. According to [65], changes in the dairy production system that are associated with the climate change are important factors, for example, economically. Therefore, researchers believe that it is essential to know the perceptions of dairy farmers about climate related risks, as this may provide a profound familiarity with susceptible nature of farmers and their adaptation behavior.

Dairy farmers have brilliant memories of the events which cause severe damage to their farming system, as mentioned in some earlier studies [26,66,67]. Older farmers can easily relate the climate change to production changes. Some national studies [39,68,69,70] explained that farmers’ perceptions are based on past disaster experiences and information of predicted climate change scenarios. Farmers’ understanding of climate change could be seen in this context. Almost all dairy farmers in Pakistan exceptionally observe climate change and its variability. This reality advocates that farmers’ knowledge of climate change and their experience of extreme weather events can improve their perception [71,72]. 

In general, findings of this study confirm that farmers have good knowledge about climate change and its variability at farm level and their perceptions are similar according to the severity and frequency of floods [73] and sometimes are more accurate with the trends of temperature and precipitation [74].

### 4.2. Perception of Climatic Risks on Dairy Production System

Keeping in view the awareness level of farmers about climate variability, researchers further tried to find out that how farmers perceive the risks against dairy production system. In South Asian countries, the huge population (25% of Global Population) of dairy animals is exposed to climate change, but it has not been recorded properly [75]. Furthermore, farmers in these countries believe that indigenous breed of cattle and buffalo have the natural ability to adjust themselves with hot and humid environment. Although several researchers have focused their intentions on the biological functions of dairy animals and found that climatic factors like heat waves have more vulnerable effects [18,51,76,77]. In this study, farmers understand the nature of climate related risks by observing productivity and changes in physical conditions of animals. Dairy farmers are very experienced to identify the impact of climate related risks on dairy animals through their observation, as some of them quoted that “animals do not tell a lie”. They can easily observe some special signs that appeared in animals to identify either they are sick or trapped by heat stress because the “body language of animals always speaks to them”. Farmers are more certain to report that which particular aspect of dairy production system is affected by climate related risks that ultimately leads to enormous damage their farm productivity as well as their revenues. Feeding and animal’s health are the two major aspects of the dairy production system that are highly affected by climate-related risks. On the other hand, milk quantity and quality is severely diminished due to pests and infectious diseases, droughts and heat waves.

Climate-related risks enhance the probability of vulnerable feeding process of animals and affect the feed quality and quantity. Farmers perceive that feeding is the main and most expensive element of dairy farming, therefore many researchers have tried to study it [78,79,80]. Fodder production is severely decreased due to increasing temperature, high CO_2_ intensity and pests and diseases. In many tropical regions, length of fodder production period is also reduced accompanied by more significant changes in precipitation patterns and more frequent droughts [81]. Due to the limited land available for small farmers in Pakistan, the provision of pasture and fodder availability is becoming problematic day by day [9]. Some farmers purchase additional feed supplies from market, but most of them are not able to afford it. Local research focusing on the feed quality, availability and improvement in feeding methods for dairy animals is either very limited or under-reported.

Climate change has an adverse effect on livestock’s health conditions in direct and indirect ways. Vector-borne diseases and factors associated with soil, floods and humidity has directly impacted animal health. Furthermore, [82] and [83] found that there was indirect effect of climate change on animal’s health, because climate change encourage the adaptation of microbes that spread the vector-borne diseases and reduced the animal’s immunity against infectious virus due to water and fodder shortages. Floods are considered severe threat, because when animals are exposed to wet and submerged pastures, probability of occurrence of parasitic liver disease is increased [8,37]. About 3000 animals perished and 2 million animals were treated by vaccination in the 2014 drought in Thar-Parkar, in a remote area of Sindh province in Pakistan [84]. 

Heat waves are ranked one of the important climate related risks due to severe climatic conditions in region. Whenever asked about heat waves, farmers mistakenly mixed their perception because heat waves are mainly observed during droughts. They also recalled that milk quantity and quality is always affected by heat waves during the last decade [8,16,25,79,85,86]. More than 120 respondents confirm that breeding is highly affected by heat waves, humidity and drought period. Most of them opined that in the dry season, the fertility of cattle is reduced, while semen quality in bulls increased in silent heat. They also considered the poor conception rate as one of the major reasons for the slow breeding process [7,37].

Although climate related risks such as heat waves, humidity, pests and diseases and drought affect the dairy farming system severely, but indigenous breeds are more adaptive to climate change. In short, farmers perceive that climate related risks have adverse impact on production system of animals.

### 4.3. Impact on Milk Production

To identify which climatic risk severely affect the milk production at farm level according to farmers’ perception, we included six climatic parameters: drought, flood, heat waves, humidity, pests and diseases and heavy rainfalls. The estimated results showed that dairy farmers perceive drought as the major climatic risk, followed by heat waves, pests and diseases, flood and humidity. The frequency of droughts and floods vary substantially with change in the frequency and intensity of rainfall. [18] concluded that due to the persistent drought conditions, the lactation period of dairy cattle’s shrinks and milk production quantity and quality also declined. In a study from Tanzania about 95% cattle owners were worried about unpredicted rainfalls as well recurrent drought periods which adversely affect the milk production and growth in cattle [87].

Heat stress in livestock due to rising temperature lead to an adverse impact on milk production [10,11]. Similar results were determined by other authors that milk production decreased due to increasing temperature and humidity [15,16,37,88]. An Indian study [89] estimated that due to different diseases milk production and weight of cattle reduced by 46% and 9%, respectively. Some other studies also found that farmers’ perceiving higher degree of severity about diseases and parasite occurrence which reduced milk production at farm level [90,91]. According to [15,16] and [17], air temperature, humidity and wind speed have significant direct effect on milk production and reproduction rate. Whenever floods occur it severely damaged the agricultural productivity (crops) and livestock sector especially. In 2010, flood in Pakistan spread over 132 thousand square kilometers damaged the livestock sector of worth US$ 441 Million [92]. The overall economic losses in livestock sector in 2014 flood were estimated about 0.23 billion rupees.

Two out of three variables of farmer characteristics showed significant perceived impact on milk production. Both education and farming experience had positive significant effects, whereas age had insignificant impact. The estimated results suggested that highly educated farmers show better understanding of climatic risk perception and its impact on production. Several studies described the role of education to combat the adverse impact of climatic risks [68,93]. We also determined in the study that dairy farming experience and age had contrasting impact on perception of farmers. The older and less experienced farmers perceived more impact of climatic risks on their dairy production system. Our results contradicted with [94] who considered the age variable as a proxy for farming experience. Our results are consistent with [8,50,95] as they concluded that experienced farmers perceive impacts of climate change in true spirits. Besides, another study [96] pointed out that age was not the best predictor of awareness for climatic variability and experience was weakly correlated with age.

For perceiving the climatic risks impacts, some farm topographies had better linkage, such as the number of milking animals and breed of animals. Farmers’ with more milking heads perceived higher impacts of climatic risks. A relevant conclusion was reported in Philippine study [8]; that herd size affected by different climatic risks, particularly drought and floods affected the larger farms. Other study from East Africa had contradicting results, because they estimated that farmers with larger herd size had better capacity and adaptation strategies against climatic hazards impacts [97]. The crossbreed cattle were found to be more sensitive than local and mixed breed; an Indian study verified that temperature and humidity affected the lactation length and lactation yield of milk about 46.50% and 48.70% in ‘Holdeo’ crossbreed cattle [86]. While our study results for breed of animals were insignificant as in Pakistan farmers prefer local breed than crossbreed.

Farmers’ linkage with institutions had greater impact on perception level. Two (source of information and contact with extension services) out of three variables in this category significantly perceive the climatic risks impacts on milk production. Respondents who had quick and reliable sources of information, perceive more impact than those who have less information or outdated information about climate changes. Many studies estimated the similar results that weather information had a bigger role in shaping the farmers’ perception about climate change [8,98,99]. Frequently contact with extension services also enhance the perception of farmers for climatic impacts on their farms as well their livelihoods. Extension services disseminate the accurate and timely information about weather and also suggests appropriate measure (according to farmers’ financial status) to mitigate the adverse impact of climatic changes. Several researchers around the world reached at the same conclusion that provision of extension services improve resilient nature of farmers against risks [49,100,101,102,103]. To conclude, the farmers who perceived higher impacts of climatic changes, also had higher intent to adapt.

## 5. Conclusions

This study analyzed the climatic risks, farmers’ perceptions, and perceived impacts on dairy farming systems, specifically in terms of milk production and risk coping adaptations practiced by the dairy farmers. Clearly evident is that dairy farmers have good awareness about climate change and its relation to extreme weather events. Most of the farmers observed that the frequency and severity of climate related risks such as droughts, floods, heat waves, pest and diseases and humidity has increased in the study area and reflects their perceptions according to the actual trends of temperature and precipitation. Farmers perceived that some climatic events like heat waves, drought, pests and diseases, floods and humidity affected dairy production system severely. However, drought was considered the most dangerous climatic risk as it affected the feeding, herd size, milk production and production cost severely. An ordered probit model showed that most of the farmers perceived that climatic risks reduced the milk production by 20% to 30%. Similarly, their income level is also affected due to reduction in milk production. To sustain their livelihoods and production, farmers adopted some risk coping adaptation strategies. More than 50% of sampled farmers changed their cropping pattern for fodder production, practice migration and tried to maintain the farming activities as well as their livelihoods needs, they do off-farm work and sell their diseased/weak animals.

At present, Pakistan’s agricultural policy focuses on improving the resilient crop production systems against climate change. This research suggests that farmers’ reliance on livestock sector is increasing, so, policies should be designed in such way that could help the livestock farmers, especially the dairy farming system, and these policies must be long term climatic resilient and locally adaptable, particularly for small scale farmers. However, the provincial government must focus on the education of farmers with the collaboration of agricultural educational institutes as well research organizations, which facilitate the farmers with short courses relevant to livestock development. Finally, this study also suggests that government should enhance the role of extension services (training programs, exhibitions of model farms, farmer to farmer contact, etc.) and media (social media, TV programs and mobile phone applications) as they motivate farmers to adopt measures against climatic risks. 

## Figures and Tables

**Table 1:** Number of Dairy Animals in the Study Area (millions).

**Territory**	**Cattle**	**Buffaloes**	**Sheep**	**Goats**	**Major Crops**
Pakistan	47.8	40.0	30.9	76.1	
Punjab	13.204	16.019	4.942	17.392	
Muzaffar-Garh (from irrigated zone of Punjab)	1.1	0.9	0.5	1.3	Cotton, wheat, maize, sugarcane
Jhang (mixed cropping zone of Punjab)	0.9	1.2	0.4	1.0	Sugarcane, cotton, wheat, maize, tobacco
Jhelum (rain-fed zone of Punjab)	0.1	0.09	0.013	0.13	Wheat, rice, fruit, vegetables, fodder

Source: Pakistan Economic Survey 2018–19 [22]; Punjab Development Statistics 2017 [44].

**Table 2:** Summary statistics of surveyed dairy farmers (*n* = 450).

Variables	Mean ± SD or *n* (%)
Socio-economic characteristics	
Age (years)	45.1 ± 12.6
Dairy Farming Experience (years)	21.2 ± 11.7
Size of Family (no. of heads)	8.5 ± 3.6
Share of Dairy Income (%)	42.8 ± 15.5
Educational level (years)	
No Education	88 (19.6)
Primary School	137 (30.4)
High School	174 (38.7)
College/University	51 (11.3)
Farm characteristics	
Farm Size (ha)	2.9 ± 1.2
Land Allocated to Dairy Animals (ha)	0.2 ± 0.01
No. of Milking Animals (no. of heads)	4.5 ± 2.6
Milk Production/per day (liter)	14.1 ± 6.5
Farm type (in number)	
Irrigated	234 (52)
Rain-fed	210 (46.7)
Mixed	6 (1.3)
Breed of dairy animals	
Indigenous	172 (38.2)
Cross	49 (10.9)
Mixed	229 (50.9)

**Table 3:** Model results with marginal effects.

Variables	Coefficients	Marginal Effects			
		Prob (Y = 1|X)	Prob (Y = 2|X)	Prob (Y = 3|X)	Prob (Y = 4|X)
		dY/dX	dY/dX	dY/dX	dY/dX
**Climatic Parameters**					
Drought	0.1404 (0.064) **	−0.014(0.007) **	−0.040(0.018) **	0.021(0.010) **	0.035(0.014) **
Flood	0.1461 (0.063) **	−0.014(0.006) **	−0.041(0.017) **	0.022(0.001) **	0.031(0.013) **
Heat Waves	0.1515 (0.065) *	−0.015(0.006) **	−0.015(0.006) **	0.023(0.011) **	0.023(0.011) **
Humidity	0.1433 (0.066) **	−0.014(0.007) **	−0.039(0.018) **	0.021(0.010) **	0.031(0.014) **
Pest & Diseases	0.1530 (0.063) **	−0.015(0.006) **	−0.043(0.016) **	0.023(0.011) **	0.032(0.013) **
Heavy Rainfall	0.0994 (0.064) ^ns^	−0.001(0.006) ^ns^	−0.028(0.017) ^ns^	0.015(0.001) ^ns^	0.021(0.014) ^ns^
**Farmers’ Characteristics**					
Education	0.0275 (0.013) **	−0.030(0.010) **	−0.080(0.040) **	0.040(0.020) **	0.060(0.030) **
Age	0.0113 (0.007) ^ns^	0.0113 (0.007) ^ns^	−0.003(0.002) ^ns^	0.002(0.001) ^ns^	0.002(0.002) ^ns^
Dairy Farming Experience	0.0201 (0.008) **	−0.002(0.000) **	−0.005(0.002) **	0.008(0.004) **	0.009(0.005) **
**Farm Characteristics**					
Milking Animals	0.026 (0.014) ***	−0.003 (0.001) ***	−0.007 (0.004) ***	0.048 (0.024) ***	0.060 (0.030) ***
Breed of Animals	0.122 (0.080) ^ns^	−0.012 (0.008) ^ns^	−0.034 (0.022) ^ns^	0.020 (0.012) ^ns^	0.030 (0.020) ^ns^
**Institutional Link**					
Social Participation	0.055 (0.072) ^ns^	−0.005 (0.007) ^ns^	−0.015 (0.020) ^ns^	0.008 (0.010) ^ns^	0.012 (0.012) ^ns^
Source of Information	0.1061(0.046) **	−0.010 (0.004) **	−0.030 (0.013) **	0.016 (0.007) **	0.023 (0.001) **
Contact with Extension	0.232 (0.080) *	−0.022 (0.008) ***	−0.064 (0.023) *	0.034 (0.013) *	0.050 (0.017) *
Services					
μ_1_			2.4518 (0.494) ***		
μ_2_			3.7753 (0.496) ***		
μ_3_			5.2023 (0.518) **		
μ_4_			6.8030 (0.576) **		
Observations			450		
LR chi2(9)			94.90		
Prob > chi2			0.0000		
Log likelihood			−512.1638		

Standard Errors are given in parentheses; *, **, *** are 1%, 5% and 10% level of significance, ‘ns’ indicates not significant.

## Appendix A

The specified model is given in Equation (A1).

(A1) $$M{\dot{P}}_{i}=\overset{14}{\underset{i=1}{\sum }}X_{i}\beta \;+\;\epsilon_{i}\;\;\;\;\;\;\;\;\;\;\;\;\;\;\;\;\;\;i=1,\;2,\ldots \ldots \ldots ..,\;n\;$$

MP*_i_*^*^ is the latent unobserved variable that corresponds perceived milk production affected by climate related risks, *X_i_* is the vector of observed explanatory variables (climatic parameters, farmers’ and farm characteristics and institutional links) of the *i*th farmer, the unknown parameter is estimated as β while the random term of the latent utility function is denoted as ϵ_i_.

The details of the model are given in supplementary section.

MP*_i_*^*^ is unobservable and we therefore observe:

$${\mathrm{MP}}_{i}\;=\{\begin{array}{c} 1\;if\;\;\;\;\;\;\;\;\;\;\;\;\;MPi*\leq \;\mu 1\\ 2\;if\;\mu \;1\;<\;MPi*\;\leq \;\mu 2\\ \;3\;if\;\mu \;2\;<\;MPi*\;\leq \;\mu 3\\ 4\;if\;\mu \;3\;<\;MPi*\;\leq \;\mu 4\\ 5\;if\;\;\;\;\;\;\;\;\;\;\;\;\;\;\;\;MPi*\;\geq \;\mu 4 \end{array}.\;$$

where MP_*i*_ = when farmers perceive ‘no impact’ ‘low’ ‘medium’ ‘hh’ or ‘very high’ by observing the statement that milk production is affected by climate change at farm level.

The unknown parameters μ_s_ are jointly assessed with β-coefficients. It is assumed that the random term of the ordered probit model is trailed as standard normal distribution. The maximum likelihood estimation methods is used to estimate the model with the probability specified as follows;

(A2) $$\begin{array}{c} P\left(MP=1|X\right)=F\left(-\beta Xi\right)\\ P\left(MP=2|X\right)=F\left(\mu 1-\beta Xi\right)-F\left(-\beta Xi\right)\\ P\left(MP=3|X\right)=F\left(\mu 2-\beta Xi\right)-F\left(\mu 1-\beta Xi\right).\;\\ P\left(MP=4|X\right)=F\left(\mu 3-\beta Xi\right)-F\left(\mu 2-\beta Xi\right)\\ P\left(MP=5|X\right)=F\left(\mu 4-\beta Xi\right)-F\left(\mu 3-\beta Xi\right).\; \end{array}$$

where F is the cumulative standard function with normal distribution. P is the likelihood of farmer for choosing either ‘no impact’, ‘low’, ‘medium’, ‘high’ or ‘very high’ with the help of given X variables.

X is vector of independent variables that affect the perception levels of farmers.

β is vector of unknown parameters to be evaluated.

### Determination of Marginal Effects

Ordered probit model narrates that as independent variables change, the coefficients from the regression are not the marginal changes in dependent variables. Therefore, it is a non-linear regression model. Marginal effects are calculated to evaluate the marginal changes. In this case, the marginal effects can be figured out as follows;

(A3) $$\begin{array}{c} \frac{\partial P\left(MP=1|X\right)}{\partial Xj}=f\left(\mu 1-\beta Xi\right)\beta j\\ \frac{\partial P\left(MP=2|X\right)}{\partial Xj}=f\left(\mu 1-\beta Xi\right)\beta j-f\left(\mu 2-\beta Xi\right)\beta j\\ \frac{\partial P\left(MP=3|X\right)}{\partial Xj}=f\left(\mu 2-\beta Xi\right)\beta j-f\left(\mu 3-\beta Xi\right)\beta j\\ \frac{\partial P\left(MP=4|X\right)}{\partial Xj}=f\left(\mu 3-\beta Xi\right)\beta j-f\left(\mu 4-\beta Xi\right)\beta j.\;\\ \frac{\partial P\left(MP=5|X\right)}{\partial Xj}=f\left(\mu 4-\beta Xi\right)\beta j-f\left(\mu 5-\beta Xi\right) \end{array}$$

where *f* (.) is a density function of standard normal variable.

$\frac{\partial P\left(.\right)}{\partial Xj}=$. The change in dependent variable given X as independent variable of the jth probability

(A4) $$\begin{array}{cc} {\mathrm{MP}}_{i}^{*(\mathrm{impact})}\;=\; & \beta_{1}^{\mathrm{op}}X_{1}^{\mathrm{op}}\;+\;\beta_{2}^{\mathrm{op}}X_{2}^{\mathrm{op}}\;+\;\beta_{3}^{\mathrm{op}}X_{3}^{\mathrm{op}}\;+\;\beta_{4}^{\mathrm{op}}X_{4}^{\mathrm{op}}\;+\;\beta_{5}^{\mathrm{op}}X_{5}^{\mathrm{op}}\;+\;\beta_{6}^{\mathrm{op}}X_{6}^{\mathrm{op}}\;+\;\beta_{7}^{\mathrm{op}}X_{7}^{\mathrm{op}}\;+\;\beta_{8}^{\mathrm{op}}X_{8}^{\mathrm{op}}\;+\;\\ & {\beta_{9}}^{\mathrm{op}}{X_{9}}^{\mathrm{op}}\;+\;\beta_{10}{\;}^{\mathrm{op}}{X_{10}}^{\mathrm{op}}\;+\;\beta_{11}{\;}^{\mathrm{op}}{X_{11}}^{\mathrm{op}}\;+\;\beta_{12}{\;}^{\mathrm{op}}{X_{12}}^{\mathrm{op}}\;+\;{\beta_{13}}^{\mathrm{op}}{X_{13}}^{\mathrm{op}}\;+\;{\beta_{14}}^{\mathrm{op}}{X_{14}}^{\mathrm{op}}\;+\;\mu_{i} \end{array}$$

where MP*_i_*^*(impact)^ = Perceived impact on milk production: 1 with no impact, 2 with low, 3 with medium, 4 with high or 5 with very high impact. X_1_ =Severity of Drought, 1 less/not severe, 2 not sure/normal severe and 3 extreme severe; X_2_ = Severity of Flood, 1 less/not severe, 2 not sure/normal severe and 3 extreme severe; X_3_ = Severity of heat waves, 1 less/not severe, 2 not sure/normal severe and 3 extreme severe; X_4_ = Severity of Humidity, 1 less/not severe, 2 not sure/normal severe and 3 extreme severe; X_5_ = Severity of pest and disease, 1 less/not severe, 2 not sure/normal severe and 3 extreme severe; X_6_ = Severity of Heavy rainfall, 1 less/not severe, 2 not sure/normal severe and 3 extreme severe; X_7_ = Education (years); X_8_ = Age (years); X_9_ = Dairy Farming Experience (years); X_10_ = Milking Animals (No. of Heads); X_11_ = Breed of Animals (1 Indigenous/local breed, 2 Mixed breed and 3 Cross breed); X_12_ = Social Participation: Member of village committee or member of any farmers’ organization/milk cooperative society/ political/NGO (1 never participation, 2 sometimes and 3 frequently; X_13_ = Sources of information about weather related (1 from extension office, 2 friends & relatives, 3 Newspaper, 4 TV and 5 Internet/Mobile); X_14_ = Contact with extension services: contact with veterinary officer/extension officer (1 Never, 2 sometimes and 3 frequently) β_s_ = Coefficient Parameters to be estimated; μ = Error term.
